# Sedimentary environment and depositional evolution of the Mesoproterozoic Bingmagou Formation on the southern margin of the North China Craton

**DOI:** 10.1038/s41598-018-26622-y

**Published:** 2018-05-29

**Authors:** Liang Yue, ZiLiang Liu, Yongsheng Ma

**Affiliations:** 1 Institute of Sedimentary Geology, Chengdu University of Technology, Chengdu, 610059 China; 2 College of Energy, Jiangsu Vocational Institute of Architectural Technology, Xuzhou, 221116 China; 3 College of Energy, Chengdu University of Technology, Chengdu, 610059 China; 4 China Petrochemical Corporation, Beijing, 100728 China

## Abstract

The Precambrian sedimentary strata on the southern margin of the North China Craton are well developed and widely exposed, making the region ideal for the study of depositional processes. However, because of the length of the depositional history and the lack of biogenic criteria, interpretations of the sedimentary environments of the Precambrian strata are often based on the tectonic background, geographical environment, rock type and sedimentary structures, resulting in controversies in the literature. In this study of the Bingmagou Formation in the Ruyang Group on the southern margin of the North China Craton, analysis of petrologic features, palaeocurrents and sedimentary facies is combined with regional correlation of relevant strata and the reconstruction of ancient landforms to explain the depositional environments and environmental transitions. Dominated by marine deposits on the southern margin of the North China Craton, the sedimentary strata of the Ruyang Group unconformably overlie the Archean crystalline basement or Proterozoic Xionger Group. As the lowermost unit of the Ruyang Group, the Bingmagou Formation, which was depositionally controlled by topography and faults and received abundant detrital material, is a highly distinctive set of sedimentary strata and represents an environmental transition from alluvial fan to sandy coast.

## Introduction

The North China Craton (NCC), one of the oldest cratonic plates on Earth, with rocks dating back to 3800 Ma, has long been a research focus of Precambrian geology in China^1–9^. The basement of the NCC has experienced several tectonic events and eventually became a component of a larger plate via collision. Although the history of inter-block rifting and collision remains disputed, the complete assembly at 1.85 Ga and the subsequent deposition of the stable cover are widely accepted. Because of the lack of basic palaeomagnetic and geological constraints, the precise location of the NCC in the Columbia Supercontinent remains controversial^10,11^. Zhao *et al*.^12^ and Zhao^13^ inferred that the eastern portion of the NCC and the South Indian plate were derived from the breakup of the same continent. According to high precision palaeomagnetic data from the rocks of the Xionger Group, Zhang *et al*.^14^ proposed that the NCC was once at a low latitude and adjacent to the Indian Plate, North Australia Plate and West Australia Plate.

The breakup of the Columbia Supercontinent coincided with collision-related ultra-high temperature, high-pressure metamorphic and magmatic events and the development of three troughs. The formation and breakup of a supercontinent have important influences on the sedimentary environment and tectonic background during the transition period. Hoffman^15^ suggested that continental margin rifting and continental disintegration are the most basic foundations for supercontinent establishment, and existing geological data indicate that the NCC was closely related to the Columbia Supercontinent. A complete set of records of the convergence, accretion and breakup processes of the Columbia Supercontinent has been preserved^6^. Unlike the central NCC, the southern margin of the NCC retains well-preserved Precambrian strata, making this region of high research significance.

**Figure 1:**
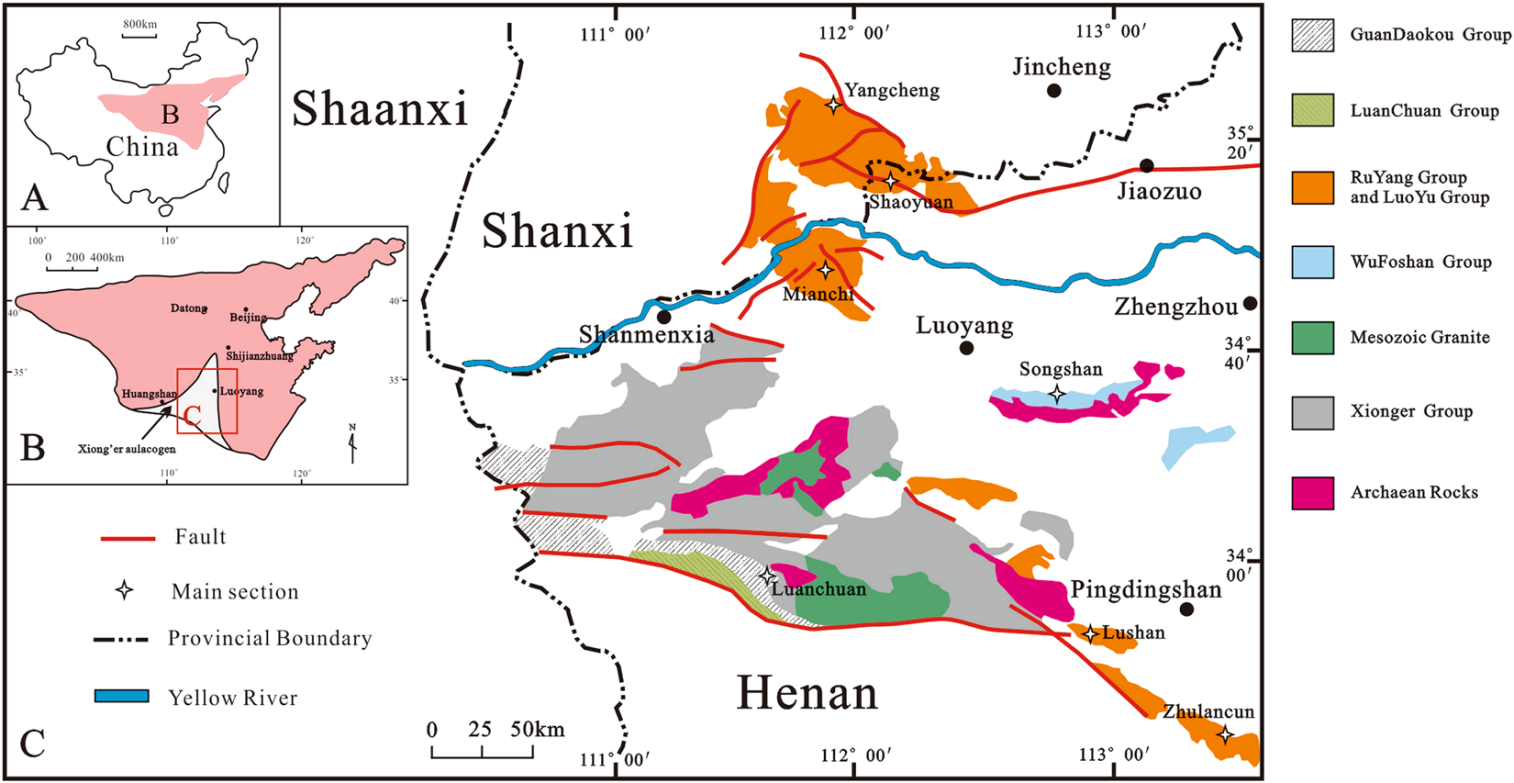
Locations of the study area and the studied sections. (**A**) North China Craton position in China; (**B**) Xionger rift trough developed on the southern margin of the North China Craton; (**C**) Geology of the study area (Fig. 1 was created and drawn with CorelDRAW X7 by Liu).

From 1.85–1.80 Ga, the NCC developed into a stable continental platform (Fig. 1A); later, however, several intracontinental faults formed. At the southern margin of the NCC, the Xionger rift trough (also known as the Shanxi - Henan rift trough) formed, with most of the trough located west of Henan Province and the central-southern portion in Shanxi Province. This trough represents the beginning of the breakup and rifting of the NCC, which occurred at a similar time to the breakup of the Columbia Supercontinent breakup (1.8–1.2 Ga)^16^. The Xionger rift trough is a three-armed rift at the regional scale (Fig. 1B), with two arms extending along the southern margin of the NCC and the third extending into central Shanxi province in a NE direction. After the formation of the Xionger rift trough, tectonic activity along the southern margin of the NCC decreased, and the region entered a stable sedimentary stage. This interval of stable sedimentary formation corresponded to a sedimentary facies transformation from terrestrial to marine from the Early Palaeoproterozoic to the Late Proterozoic.

The Chattisgarh Supergroup developed on the Indian plate^13,14^, which was adjacent to the NCC during the Mesoproterozoic and underwent a similar sedimentary and tectonic evolution. A transition from an alluvial fan to a high energy continental shelf environment has been identified in these rocks^17^. Like the Xionger rift trough, the Rjukan rift basin on the passive continental margin of southern Norway in the Mesoproterozoic developed typical alluvial fans under the control of ancient landforms, climate and structures^18^. In the early Mesoproterozoic, a global transgression event occurred and was closely related to the breakup of the Columbia Supercontinent.

Based on field geological investigations, petrologic observations, sedimentary facies analysis and palaeogeographic study, the depositional genesis of the Bingmagou Formation has been analysed, and the palaeogeomorphology, depositional setting, material sources and tectonic evolution of the southern margin of the NCC during the Early Proterozoic have been investigated. This work provides a basis for the correlation and division of the stratigraphic sequences of the region and provides new evidence for the evolution of the southern margin of the NCC and Columbia Supercontinent.

## Regional geological background

Proterozoic strata are well developed and widely exposed in western Henan Province and southern Shanxi Province, making regional stratigraphic tracing and correlation straightforward. A number of stratigraphic classification schemes have been proposed^5,19,20^, but most have divided these strata into three regions: Mianchi - Queshan, Songji and Xiongershan. The Mianchi - Queshan region contains the Ruyang Group, the base of which was previously termed the Xiaogoubei Formation. The Songji region contains the Wufoshan Group, the base of which is the Bingmagou Formation. Finally, the Xiongershan region contains the Guandaokou Group, the bottom of which is the Gaoshanhe Formation. The Xiaogoubei Formation is equivalent to the Bingmagou Formation^21^ but was previously assigned a different name; hence, the use of the term Xiaogoubei Formation should be abandoned^22^.

**Figure 2:**
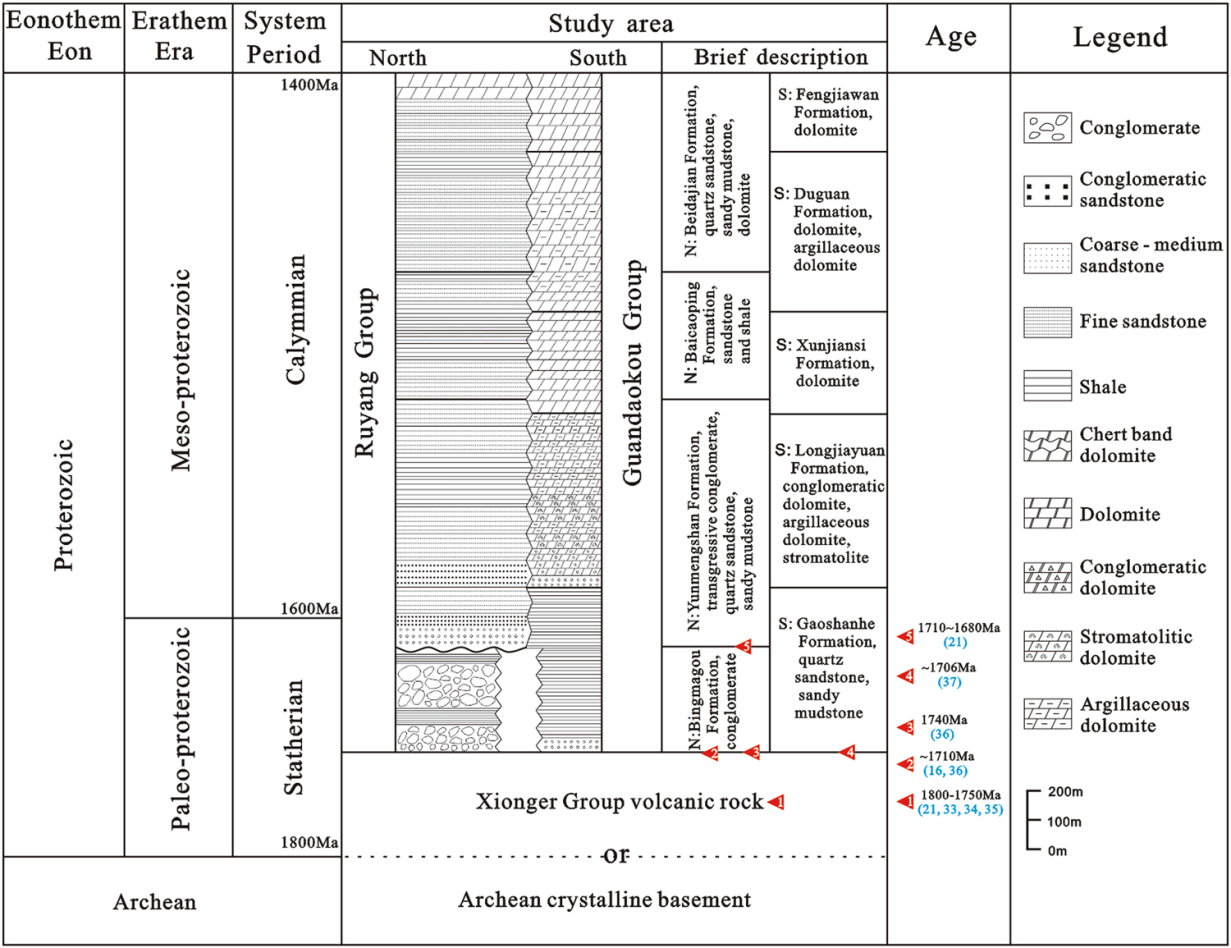
Regional stratigraphic column of the study area (21)^59,62–65^.

The Proterozoic outcrops in the study area are mainly composed of the Ruyang Group, although the southwestern Luanchuan region also contains outcrops of the Guandaokou Group (Figs 1C and 2). The Mesoproterozoic Ruyang Group, which is thousands of meters thick, is mainly composed of conglomerate, sandstone and mudstone with local limestone and dolomite. The Bingmagou Formation, the lowermost unit of the Ruyang Group, is characterized by abundant conglomeratic and sandy clasts and overlies the NCC crystalline basement or the Xionger Group. Controlled by topography and tectonism, it is limited in distribution and changes rapidly in thickness. The Bingmagou Formation only crops out in the regions of Shaoyuan, Mianchi, Songshan, and Lushan and near Zhulan village (Fig. 1C); it is absent in other regions. Previous fieldwork revealed that the Bingmagou Formation developed in the northern and eastern parts of Xionger trough, where are considered the most active regions of the ancient fault^22^. There are few previous studies of the Bingmagou Formation and Ruyang Group, and most of them generally focus on the age and microbial deposition of the Ruyang Group^1,5,23–25^. Only a few studies focus on the sedimentology and stratigraphy of the Ruyang Group^26–28^ and they largely ignore the Bingmagou Formation at the bottom of the Ruyang Group. Although the Bingmagou Formation is only exposed locally, its significance cannot be ignored, especially because it represents the beginning of the deposition of stable sedimentary cover strata on the NCC. There are several views regarding the formation of the Bingmagou Formation due to its variable thickness and outcrop discrepancies. Most researchers adhere to the view proposed in the 1970s~1980s that it was deposited in a continental environment and includes alluvial fan - fan delta facies^29^, alluvial fan - river facies^28^, piedmont - river delta - coastal facies^30^, and alluvial fan facies^31,32^.

Although the Bingmagou Formation is limited in distribution and varies significantly in thickness, it still follows a characteristic pattern. The Bingmagou Formation in the Shaoyuan region can be up to 872.1 m in thickness, the thickest in the study area, but it thins to 164.8 m in the Mianchi region to the southwest. In the Songshan region, the formation is 566 m thick but thins to 39 m in the Lushan region to the south and to 25 m in the Zhulandian region in the southernmost part of the study area. The overall topography at the southern margin of the NCC is lower in the south and higher in the north, with palaeo-uplifts in the Songshan region^33^. The Bingmagou Formation is very thick in the regions proximal to the material supplied from the palaeo-uplift, Shaoyuan, Mianchi and Songshan, but is much thinner in the Lushan region and in Zhulan village, far from the uplift area. The conglomerates in the formations in different areas are complex and contain Archaean Dengfeng Group gneiss and Palaeoproterozoic magmatic rocks and metamorphic quartzite, among other rock types. From north to south, the Bingmagou Formation thins gradually, possibly influenced by the gentler topography and limited source material, especially in the Lushan region and in Zhulan village, where the lower part of the alluvial fan deposits is missing and only marine sedimentary strata of the upper Bingmagou Formation are present. In terms of distribution, marine deposits cover the whole area, but alluvial fan deposits are distributed locally only in the Shaoyuan, Mianchi and Songshan regions^22^.

## Facies analysis and interpretation

In the present study of the Bingmagou Formation of the Ruyang Group on the southern margin of the NCC, four main sections (Yangcheng, Shaoyuan, Mianchi and Luanchuan) and three observation sections (Songshan, Lushan and Zhulan village) were selected for investigation (Fig. 1C). The Bingmagou Formation is found in all areas except for the Yangcheng and Luanchuan regions. The following sections focus on the stratigraphic sections of the Bingmagou Formation in the Shaoyuan and Mianchi regions.

Palaeocurrent information is one of the most important indicators for determining basin margin orientation, provenance location, palaeoslope direction, and sand body orientation^34,35^. In this study, palaeocurrent data were collected mainly from large inclined beds, trough cross bedding and imbricate structures in the conglomerate, following the methods proposed by High and Picard^36^ and Ramsay^37^. The results for the Shaoyuan and Mianchi regions indicate that the palaeocurrent direction is mainly NE, with a unimodal fan-shaped distribution, and the main provenance was the NCC^21,22^.

The grain-size distribution and association of clastic sedimentary rocks are good indicators of the sedimentary environment and hydrodynamic conditions. One widely used and effective method is the analysis of grain-size parameters and cumulative grain-size probability curve^38–40^. Zheng^29^ analysed the particle size distribution of siliceous clastic samples from the Bingmagou Formation and suggested that the Bingmagou Formation was deposited in an alluvial fan near its source, in conjunction with strong hydrodynamics.

**Figure 3:**
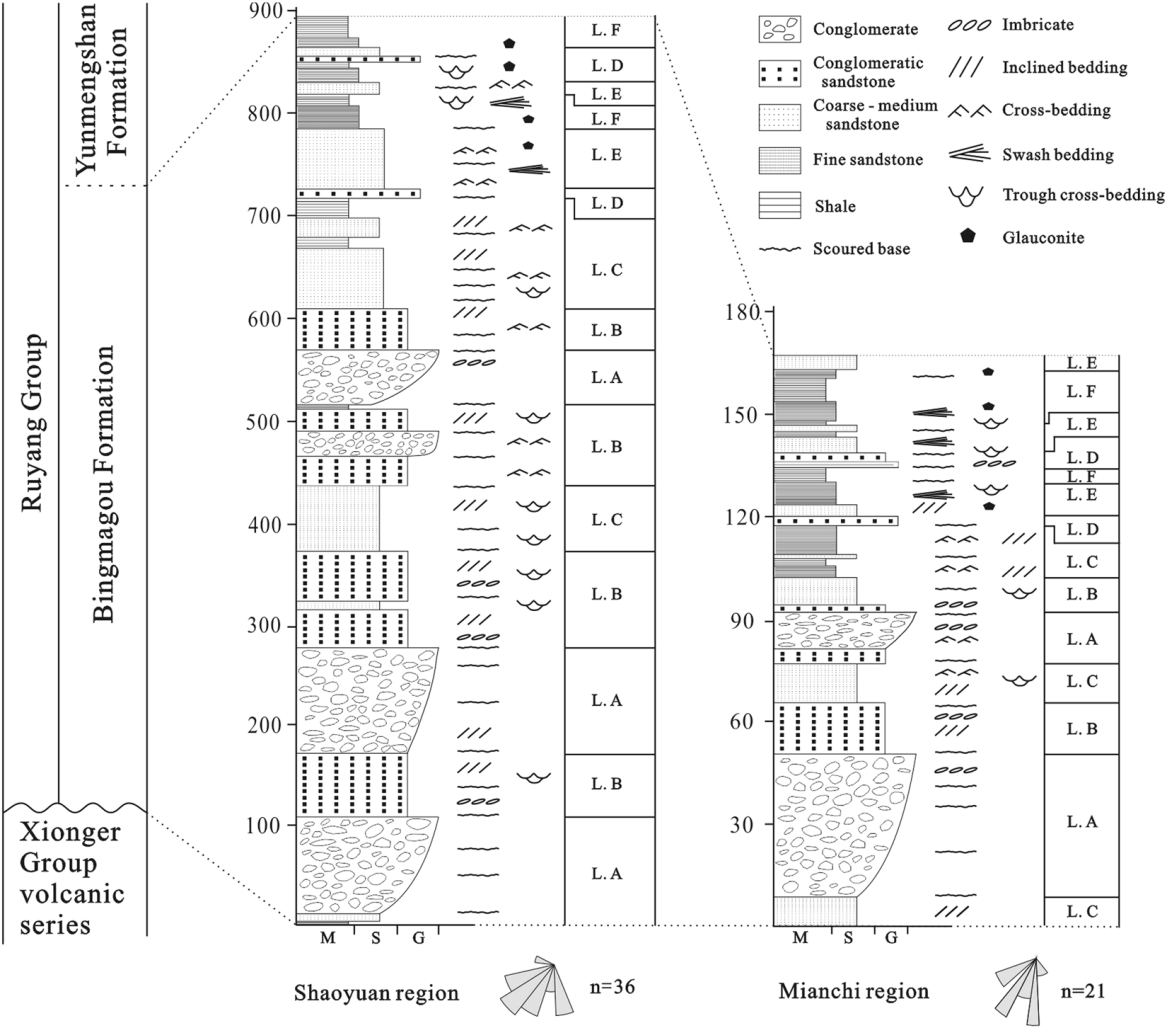
Comprehensive histogram of the Bingmagou Formation in the Shaoyuan and Mianchi regions. M = Mud; S = Sand; G = Gravel.

The Bingmagou Formation in the Shaoyuan region can reach thicknesses exceeding 870 m, the largest outcrop thickness in the study area (Fig. 3); it consists predominantly of conglomerate, conglomeratic sandstone, fine-to-coarse sandstone, siltstone and shale. Two types of sedimentary environment were identified based on lithology, sedimentary structures and palaeocurrent data, which divide the formation into two members (Table 1). The lower member includes terrestrial deposits composed of alluvial fan facies, and the upper member includes marine deposits composed of sandy coast facies. The Bingmagou Formation in the Mianchi region is 165 m thick (Fig. 3), like that in the Shaoyuan region, and it was also divided into two members based on the sedimentary environment. The lower member is composed of alluvial fan deposits with a smaller thickness and smaller gravel sizes than the lower member in the Shaoyuan region, indicating that it was closer to the alluvial fan edge, and is composed of a higher proportion of sand and mud. The upper member consists of shoreface deposits with a transgression-related basal conglomerate, above which lie typical marine deposits with widespread inclined bedding, cross bedding and swash bedding, as well as glauconite deposits.

**Table 1:** Characteristics of the lithofacies association identified in the Bingmagou Formation.

Lithofacies association	Lithofacies	Code	Description	Sedimentary environment
Terrestrial Lithofacies Association	Clast-supported and matrix-supported conglomerate facies	A	Up to 5 m thick; low maturity, poorly sorted and rare primary sedimentary structures; gravel sizes from 2–50 cm	Debris flow; fan root
	Conglomeratic sandstone - medium coarse sandstone lithofacies	B	Up to 4 m thick; gravels are complex in composition, with various sizes (1–5 cm), angular to sub-rounded, and in an imbricated arrangement locally; inclined bedding	Debris flow; the middle and upper part of alluvial fan deposits; river channel
	Fine-grained lithofacies	C	Up to 2 m thick; fine sandstone, siltstone and shale; lacking cross bedding and other sedimentary structures	Liquid flow; sheet flow; terminal fan
Marine Lithofacies Association	Transgression basal conglomerate - conglomeratic sandstone facies	D	Up to 2 m thick; shale chip gravels, with smaller sizes of 0.5–2 cm; intrastratal bedding is abundant; scoured surfaces are quite common	Transgressive deposition; nearshore
	Sandstone facies with composite bedding	E	Up to 3 m thick; extensive scoured surfaces; parallel bedding, cross bedding, swash bedding, extensive vein and lenticular sand bedding and bunchy bedding; generally interbedded with sandy shale, constituting rhythmites; glauconite and iron nodules present	Foreshore and nearshore
	Shale facies	F	Up to 2 m thick; silty and muddy deposits; glauconite; horizontal bedding	Backshore

### Terrestrial lithofacies association

The terrestrial lithofacies association, accounting for 70% or more of the main sections (Shaoyuan and Mianchi), unconformably overlies metamorphic basement or the Proterozoic Xionger Group of the NCC and occurs widely in the Shaoyuan, Mianchi and Songshan regions.

As the clastic sedimentary facies with the largest grain size, the alluvial fan deposits are mainly characterized by a thick conglomerate layer. This layer was interpreted by previous researchers as river channel sediments in the fluvial facies to account for the limited distribution and abrupt changes in thickness of the Bingmagou Formation. However, the gravel deposits with thicknesses of tens or even hundreds of metres cannot be so explained. The deep purple colour, poor sorting, low maturity, and large sedimentary thickness indicate full exposure of the clastics and accumulation near the source, which is more consistent with the sedimentary characteristics of an alluvial fan. The terrestrial lithofacies association consists of the three lithofacies described below.

#### Lithofacies A: Clast-supported and matrix-supported conglomerate facies

**Figure 4:**
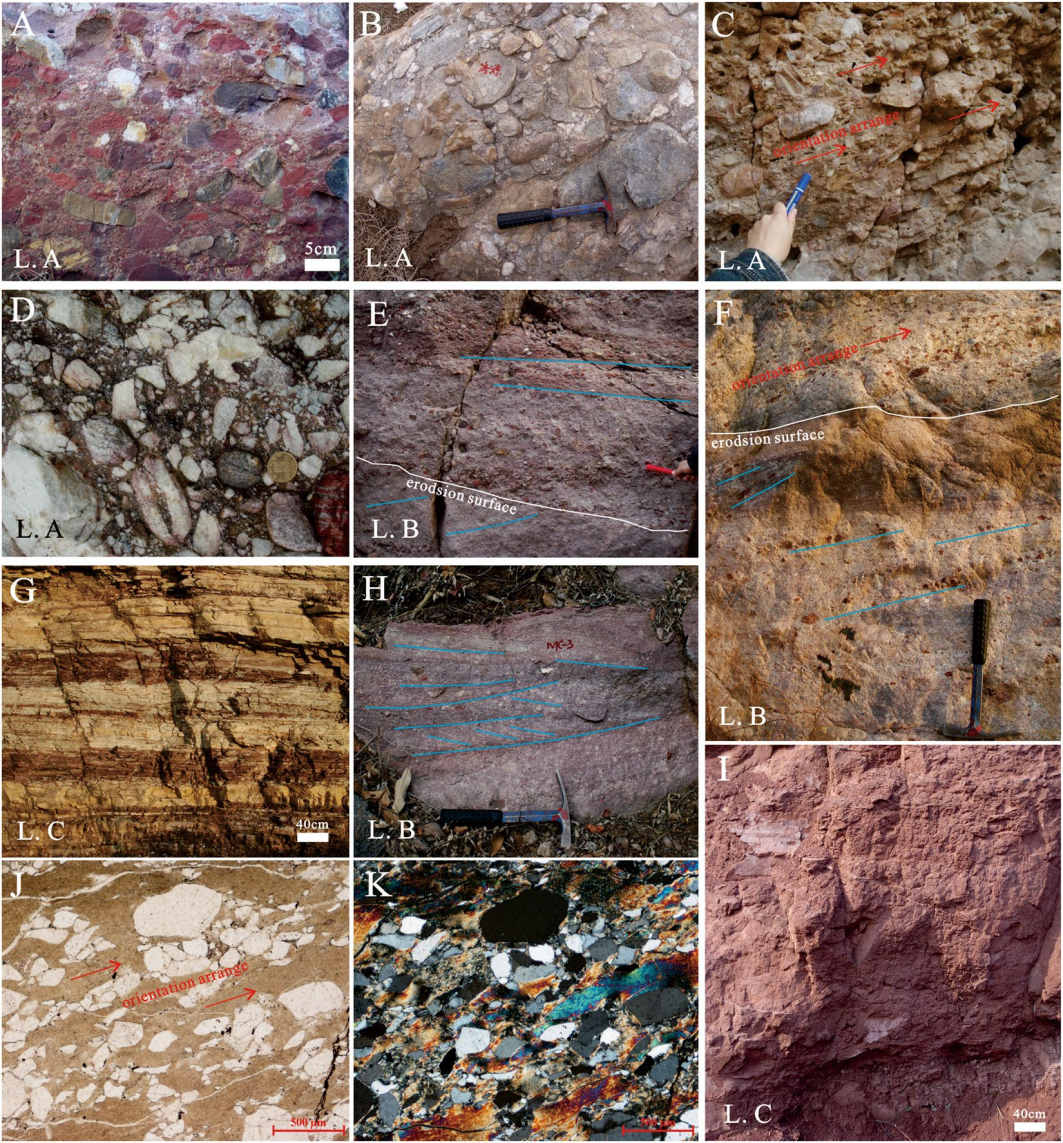
Outcrop and microscopic characteristics of Bingmagou Formation rocks in the Shaoyuan and Mianchi regions. (**A** and **B**) Grain-supported conglomerate in the Shaoyuan region, with a complex gravel composition, various grain sizes, poor sorting, poor roundness, and no obvious sedimentary structures. (**C**) Matrix-supported conglomerate in the Mianchi region, with oriented gravel clasts (long axis parallel to the bedding plane). (**D**) Grain-supported conglomerate in the Mianchi region without obvious sedimentary structures, in which gravel clasts are mostly quartzite and gneiss. (**F**) Conglomeratic sandstone in the Shaoyuan region; the conglomeratic sandstone is interbedded with layered sandstone, and the gravel clasts exhibit directional arrangement. (**E** and **H**) Conglomeratic sandstone in the Mianchi region, in which the gravels are complex in composition and arranged along the scoured surface or bedding plane. (**H**) Wedged bedding, and laminations inclined unidirectionally within a single layer. (**G**) Purple shale interbedded with greyish brown fine argillaceous sandstone in the Shaoyuan region. (**I)** Red sandy mudstone in the Mianchi region. (**J** and **K**) Microscopic characteristics of argillaceous sandstone in a matrix-supported sandstone, in which the quartz grains are directionally arranged, angular to round, and poorly sorted. (**J**) Plane-polarized light. (**K**) Cross-polarized light.

This lithofacies is characterized by a low maturity, poor sorting and rare primary sedimentary structures (Fig. 4A–D). The top and bottom interfaces of each bed are generally parallel but are locally wavy because of the development of a scoured surface. The conglomerate is greyish brown or aubergine and medium to thick (0.3–5.0 m), and the gravels vary in size from 2–50 cm, are angular to sub-rounded and are not obviously oriented. A matrix-supported conglomerate is a typical deposit of a debris flow, in which the abundance of the argillaceous matrix gives rise to a high pore pressure, causing gravel floating, and the long axis of most gravels is parallel to the bedding plane (Fig. 4C).

A debris flow generally features a steep front and an abundance of mud and coarse clastics, and its flow velocity is lower than that of the main body and may slow down, resulting in thick deposits^41,42^. Thus, the lower Bingmagou Formation alluvial fan deposits have thick sedimentary layers of conglomerate. The transitions between the grain-supported conglomerate and the overlying matrix-supported conglomerate are generally gradual, but sometimes abrupt changes occur in the transitions from the matrix-supported conglomerate to the grain-supported conglomerate.

#### Lithofacies B: Conglomeratic sandstone and medium-to-coarse sandstone lithofacies

The conglomeratic sandstone is mainly aubergine or greyish yellow and generally has thin to thick layers (0.1–2.0 m), and the gravels within the unit are complex in composition, of varying size (1–5.0 cm), angular to sub-rounded, and local form imbricated arrangements (Fig. 4E,F,H). The bottom surface of the conglomeratic sandstone layer is generally scoured, with gravels accumulated and arranged along the scoured face. Within this layer, there is inclined bedding, wedge-shaped bedding, and trough cross bedding. The long axis of most gravel clasts is parallel to the bedding plane. The vertical sequence of the conglomeratic sandstone shows normal grading, with the clastic grain size decreasing upward and transitoning to sandstone; abrupt changes to sandstone are present locally, but these changes have a limited lateral extent. The medium-coarse sandstone mainly includes two types: one transitions from the conglomeratic sandstone layer, with extensive internal oblique bedding, wedge-shaped bedding, trough cross bedding and good lateral continuity, and the other occurs in horizontal beds or lenses without obvious sedimentary structures, has rare parallel bedding with obscure, thin layers and with a short lateral extent.

This type of medium sandstone occurs mainly in the middle and upper parts of alluvial fan deposits and reflects a clastic sedimentary environment under the influence of hydrodynamic changes within the alluvial fan. When the hydrodynamics are strong, it is easy to form debris flow deposits or sandstone layers with abundant internal sedimentary structures; when they are weak, sandstone layers with no obvious stratification are formed; and when they are weaker and the water is static, fine-grained sediments are deposited, which is generally attributed to the decrease in gravity flows and reflects layered transport and the accumulation of fluids^43–45^. The formation of medium sandstone with abundant bedding structures can be attributed to highly viscous debris flows or subaqueous one-way sheet flows^46^.

#### Lithofacies C: Fine-grained lithofacies

The fine-grained lithofacies include the fine sandstone, siltstone and shale of the alluvial fan deposits (Fig. 4G,I–K). They are reddish-brown or greyish yellow in colour, with layers ranging from 20 cm to 2 m in thickness; they lack cross bedding and other sedimentary structures and have occasional horizontal bedding. Sandy rocks are often interbedded with argillaceous rocks (Fig. 4G), with the shale content increasing upwards. A large amount of fine clastic deposits represents the vertical settling of suspended sand and mud in a long-term static aqueous environment^47,48^.

Flows rich in clastics are generally composed of a base with high mechanical strength and an overlying fluid, with gravelly clastics at the base of the deposit and sandy layers along the front and sides^49^. This explains the genesis of the normally graded sedimentary sequence in an alluvial fan. Hence, fine-grained homogeneous deposits generally occur in the upper part of the depositional cycle (Fig. 3). However the top interface of the fine-grained depositional system can also change abruptly into a conglomeratic sandstone, indicating sheet flow deposits on the surface of the distal fan.

### Marine lithofacies association

Strata deposited in the shore zone generally occur in the upper Bingmagou Formation, which features a wider range of distributions than the alluvial fan deposits and crops out in the Shaoyuan, Mianchi, Songshan, and Lushan regions and in Zhulan village. In Zhulan village, the Bingmagou Formation near-shore sediments directly overlie the Xionger Group and the alluvial fan sedimentary unit is absent.

**Figure 5:**
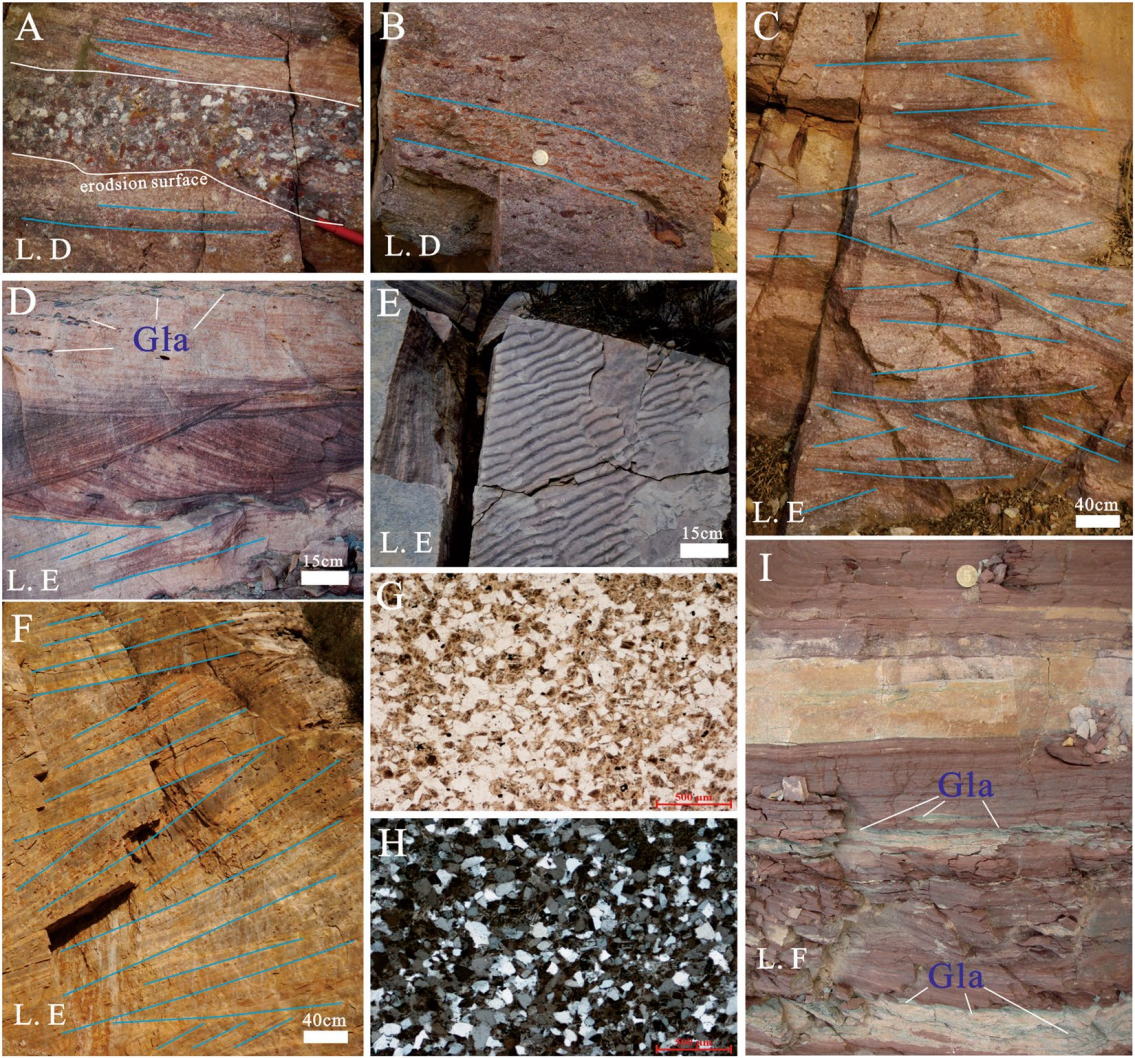
Outcrop and microscopic characteristics of Bingmagou Formation rock in the Mianchi and Shaoyuan regions. (**A**) Coarse sandstone interbedded with conglomerate; gravel clasts in the conglomerate are complex in composition (mainly quartz and muddy gravel), generally small and well rounded, but the muddy gravel clasts are moderately rounded. (**B**) Coarse sandstone with gravel; the gravel clasts are complex in composition but are mainly muddy gravels arranged directionally with the long axis parallel to the scoured surface. (**C**) Coarse sandstone with gravel and cross-bedding in the Shaoyuan region. (**D**) Fine-to-medium sandstone in the Mianchi region, with trough cross bedding, parallel bedding, and glauconite in the upper part (Gla = glauconite). (**E**) Fine sandstone in the Lushan region, with tuning fork-shaped ripple marks on sandstone surfaces. (F) Fine-to-medium sandstone in the Mianchi region, with low-angle swash bedding. (**G** and **H**) Microscopic characteristics of shoreface sandstone in the Shaoyuan region; the sandstone is rich in quartz, high in maturity, well sorted, with sub-rounded and rounded quartz grains. (**I**) Red sandy mudstone with thin siltstone interbeds and layered or lenticular glauconite.

The Precambrian coastal deposits are characterized by high maturity, well-sorted sandstone, in which the main sedimentary structures include hummocky, sunken, herringbone, trough and tabular cross-bedding and parallel bedding^50–54^. In the study area, the sandstone layers in the Bingmagou Formation have numerous typical sedimentary structures, such as composite cross-bedding, cutting-activated bedding planes (Fig. 5C,F), herringbone cross-bedding, glauconite (representing marine deposition; Fig. 5D), and tuning fork-shaped ripple marks (Fig. 5E), indicating that the upper Bingmagou Formation is a marine deposit. Based on the identified sedimentary facies, the marine lithofacies association can be divided into 3 lithofacies.

#### Lithofacies D: Transgression-related basal conglomerate and conglomeratic sandstone facies

This lithofacies is mainly composed of transgression-related basal conglomerate and conglomeratic sandstone. Unlike the alluvial fan gravelly deposits of the Bingmagou Formation, the gravels in these facies, although also complex in composition, have a larger proportion of shale chip gravels and overall smaller sizes of approximately 0.5–2.0 cm. In layers of this facies, intrastratal bedding structures are common and include inclined bedding, cross bedding and swash bedding. Scoured surfaces are also quite common, and gravels are directionally arranged along the scoured surface or lamella.

The transgression-related basal conglomerate generally overlies the sandstone or shale in the upper alluvial fan deposits, with a scoured surface commonly representing the contact surface. The gravel in this conglomerate is mostly quartz and mud. The quartz gravel represents the influx of terrigenous clasts or reworking of the original alluvial fan gravelly deposits; thus, the gravels are generally smaller in size and well rounded (Fig. 5A). The muddy gravels could be the result of transgression-related erosion and migration of the original alluvial fan sandy deposits; thus, the muddy gravels are poorly rounded, representing proximal or *in situ* deposition (Fig. 5B).

#### Lithofacies E: Sandstone facies with composite bedding

This lithofacies is mainly composed of purple or greyish yellow feldspar-quartz sandstone, quartz sandstone or argillaceous sandstone and is generally in thin or medium-thick layers. These beds feature obvious erosion interfaces, extensive scour and filling structures, parallel bedding, cross bedding, swash bedding, extensive vein and lenticular sand bedding, bunchy bedding, and interbeds of sandy shale that form a rhythmite. There are floating gravel clasts in the facies, but the clasts are small (less than 2 cm). Some of the layers contain glauconite and iron nodules, and some surfaces contain ripples.

Sediments in the shoreface and foreshore belts are prone to forming sedimentary structures because of washing and disturbance by waves action. Sediments in shore areas have simple mineral compositions, dominated by quartz with high maturities, and the surfaces feature structures like wave marks, erosional surface and crossing bedding^55^.

#### Lithofacies F: Shale facies

This lithofacies contains silty and muddy deposits (which are usually purple), mudstone, sandy mudstone and siltstone in thin or medium-thick layers. This lithofacies is generally located in the upper part of the normal grading sequence, with some units containing sandstone and conglomeratic sandstone and occasional glauconite in extensive horizontal layers.

Lithofacies F represents a relatively quiet but exposed sedimentary environment (Fig. 5I). Generally, it is interpreted as backshore deposits that were usually exposed subaerially and were only flooded at high tide or storm tide; water gathered in low-lying areas, resulting in silty or muddy deposits.

## Discussion

The Xionger rift trough and the Xionger Group on the southern margin of the NCC (approximately 1.8–1.75 Ga in age)^13,56–58^ are the products of the extension and breakup of the southern margin of the NCC during the Meso-Neoproterozoic, corresponding to the breakup of the Columbia Supercontinent. Comparisons of Precambrian strata remain controversial due to the lack of specific chronological data. Previous research has shown that the Proterozoic Ruyang Group was deposited in a passive continental margin environment and is not older than 1.75~1.60 Ga and is mainly composed of siliciclastic and carbonate deposits^6^.

The Bingmagou Formation, as the base stratigraphic unit of the Ruyang Group (overlain by the Yunmengshan Formation with a basal age of 1710 Ma)^16,59^, developed after the formation of the Xionger Group, which has been dated. Thus, the available data constrain the age of the Bingmagou Formation to 1.75~1.70 Ga (Fig. 2).

### Sedimentary evolution

Geographically, the Luanchuan, Mianchi, Shaoyuan and Yangcheng regions from south to north are in the NE branch of the Xionger rift trough (Fig. 1B,C). Closer to the centre of the Xionger rift trough and near the southern margin of the NCC, the Luanchuan region was the first area to have experienced transgression and was the area with the deepest water during the late transgression. The Mianchi, Shaoyuan and Lushan regions were, on the one hand, influenced by the higher terrain in the north and lower terrain in the south; and on the other hand they were situated in the fault zone of the Xionger rift trough and were significantly influenced by this fault zone. In these regions, there are alluvial fan sedimentary strata of the lower Bingmagou Formation, and although tectonism may not be the main factor affecting the formation of the alluvial fan^60,61^, its influence on the sedimentary characteristics, fan shape and deposition thickness cannot be neglected^56,58,60,61^.

**Figure 6:**
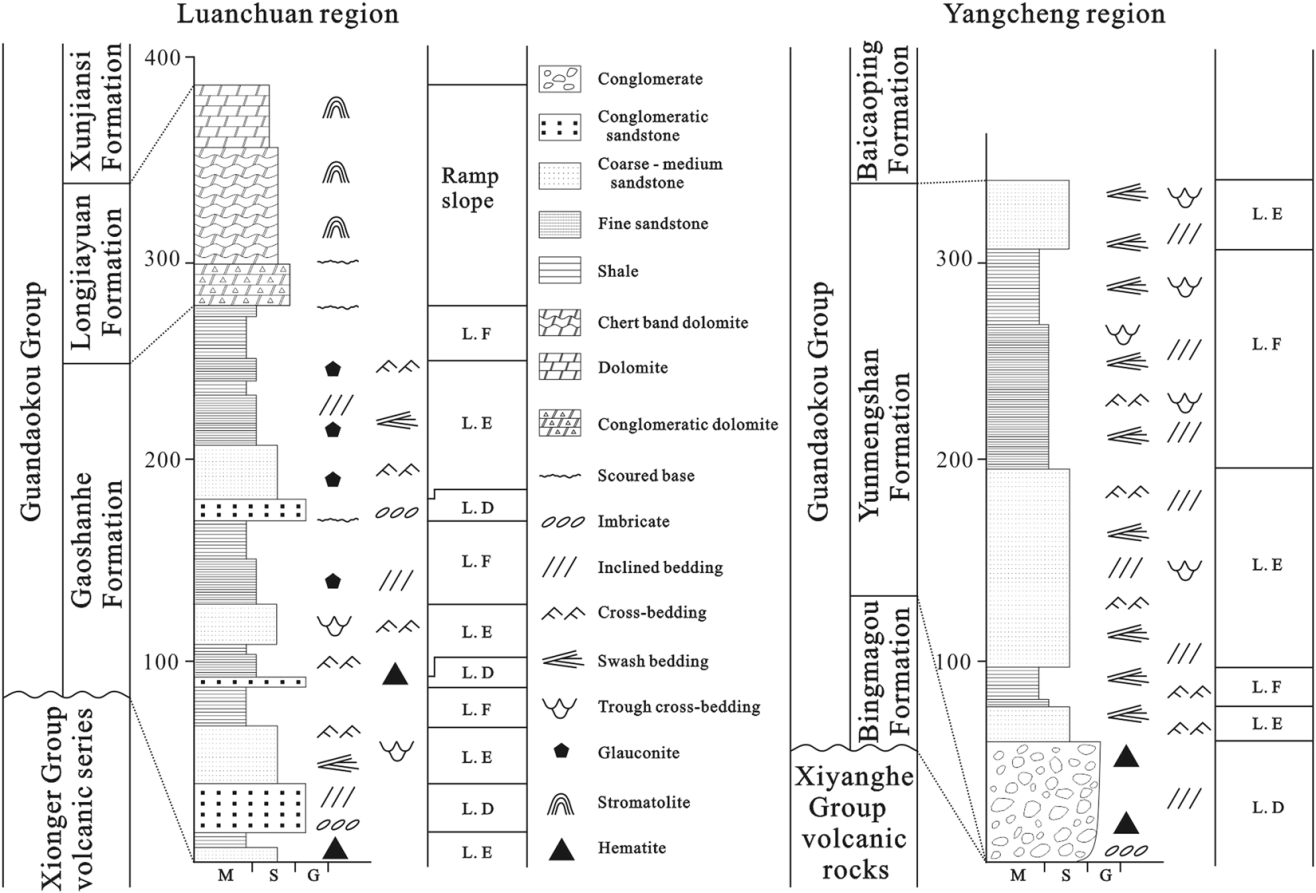
Composite columnar stratigraphic section of the Luanchuan and Yangcheng regions.

As the bottom of the Ruyang Group, the Bingmagou Formation crops out only in the Shaoyuan, Mianchi, Songshan, Lushan and Zhulan village regions; it is absent in the Yangcheng and Luanchuan regions (Fig. 6). Due to extensive gravity flows resulting from long-term faulting, the Bingmagou Formation alluvial fan resulted in thick gravelly deposits and fan-shaped deposits^19^. With the transgression, marine coastal sediments began to be deposited in the upper Bingmagou Formation. When the Bingmagou Formation was deposited, the Yangcheng region was higher and experienced weathering and erosion, thereby acting as the provenance of clastic material. The sedimentary strata of the Yunmengshan Formation above the Bingmagou Formation did not develop until the late transgression.

**Figure 7:**
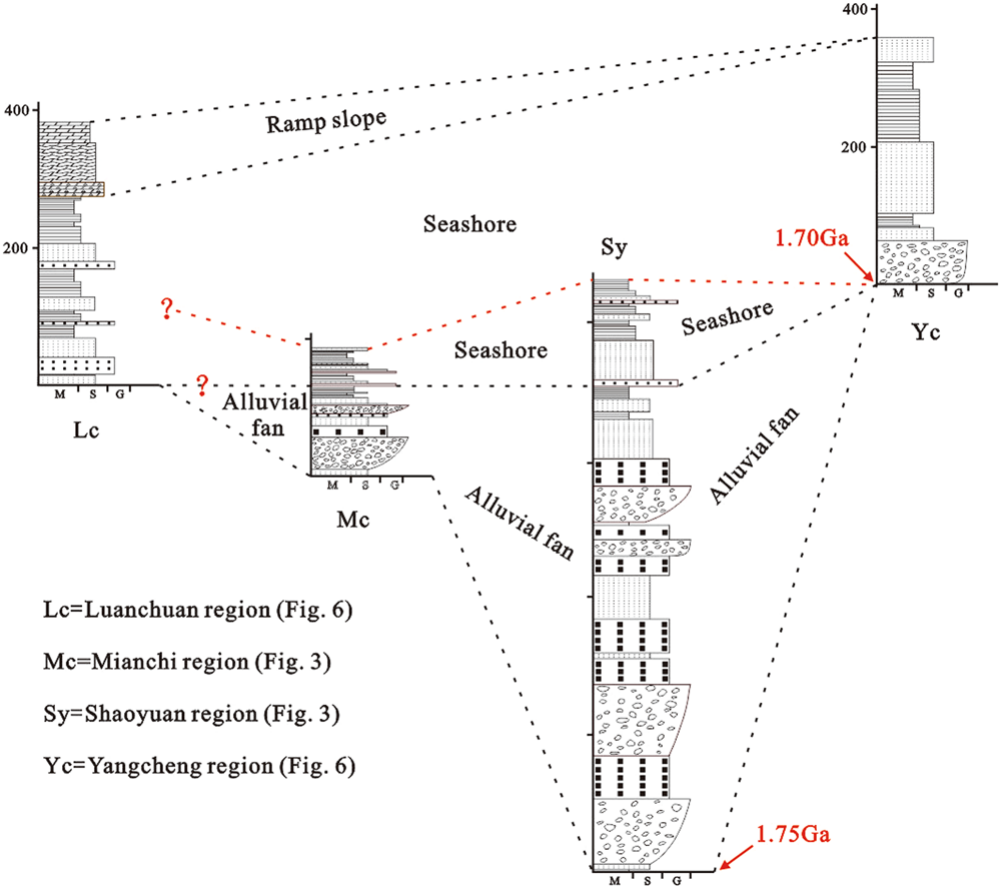
Stratigraphic correlation and sedimentary facies evolution of the Bingmagou Formation on the southern margin of the North China Craton.

With similar volcanic layers at the bottom of each, the Gaoshanhe Formation and the Yunmengshan Formation have often been correlated, but doubts remain^32,59^. Wang^59^ obtained a middle-late age for the volcanic layers. The stratigraphic correlation between the Gaoshanhe Formation and the Yunmengshan Formation is indisputable given both formations are marine deposits unconformably overlying the Xionger Group volcanic rock. However, in the area where the Bingmagou Formation occurs, could the marine strata of the upper Bingmagou Formation have been deposited at the same time as the lower Gaoshanhe Formation (Fig. 7)? Could the Gaoshanhe Formation in the Luanchuan region on the southern margin of the NCC have undergone transgression first and thus be older than the Yunmengshan Formation inland? The existence of the Bingmagou Formation indicates a short period of exposure and erosion (1.75~1.70 Ga) after the formation of the Xionger Group along the southern margin of the NCC, followed by a new sedimentary cycle.

### Sedimentary model

Compared with the research on the Yanliao rift trough, the Xionger rift trough requires further study. The sedimentary system of the Xionger rift trough is the result of tectonic events related to the breakup of the Columbia Supercontinent. The volcanics and sediments of the Xionger Group constitute the basement of the rift trough upon which the Guandaokou Group and Ruyang Group were deposited, producing a sedimentary cover.

**Figure 8:**
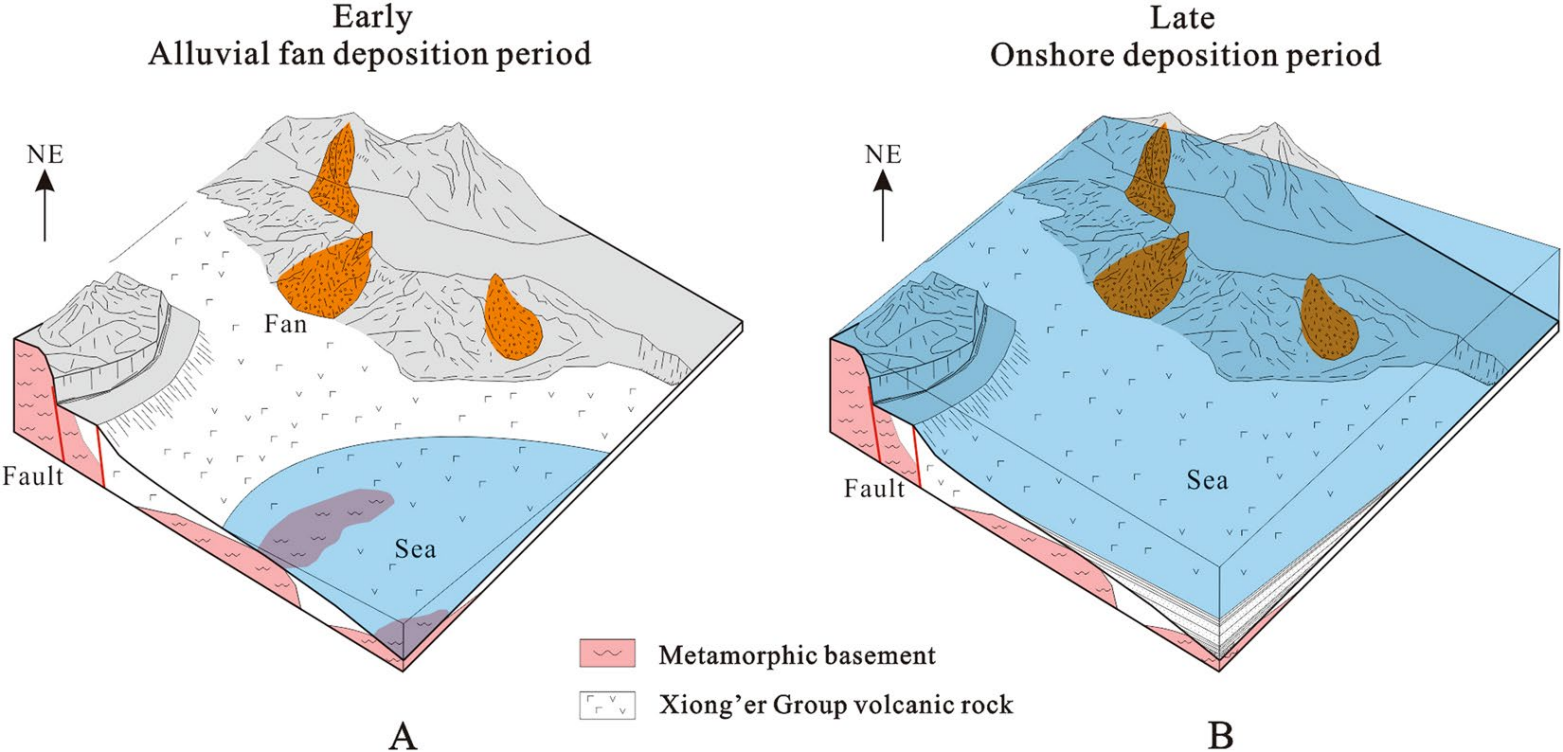
Sedimentary model of the Bingmagou Formation on the southern margin of the North China Craton. (**A**) Development period of the Bingmagou Formation alluvial fan. (**B**) Development period of the Bingmagou Formation coastal facies (Fig. 8 was created and drawn with CorelDRAW X7 by Liu).

In the early Proterozoic, seawater entered the Xionger rift trough but deposition was limited on the southern margin of the NCC. As shown in Fig. 8A, at the time the Luanchuan region was a marine coastal zone, but the Lushan, Mianchi, and Shaoyuan regions along the NE branch of the rift trough were uplifted areas. Due to the relief generated by faults, alluvial fans rapidly developed near source areas, forming thick clastic strata characterized by clasts with poor sorting, poor roundness, large average sizes and complex compositions. The alluvial fan strata of the Bingmagou Formation on the southern margin of the NCC feature small spatial distributions and radial extents, extending and thinning out from the northeast to the southwest due to the terrain being higher in the north and lower in the south.

Later, with ongoing transgression, most of the area changed from terrestrial to marine deposition, and coastal sedimentary strata of the upper Bingmagou Formation developed (Fig. 8B). The identifiable transformation interface from the alluvial fan to a coastal environment is the bottom of the transgressive lag conglomerate layer. In the coastal facies deposits, the conglomerate layer is thinner, the gravels are smaller in size and better rounded, and there is typical bi-directional cross bedding, bunchy bedding and swash bedding in the marine sandstone above, which differs from the unidirectional bedding in the underlying alluvial fan deposits. Furthermore, ripple marks and glauconite are abundant in the sandstone and mudstone layers. In addition, the coastal sedimentary strata have a wider spatial distribution, and they can be traced and correlated across the region.

In general, the alluvial fan sedimentary strata in the lower Bingmagou Formation have a smaller distribution range than the coastal sedimentary strata, since the alluvial fan deposits were strongly controlled by structures and the terrain, and as a result are distributed locally. The marine deposits that formed during the transgression are much more extensive than the earlier alluvial fan deposits, resulting in the local development only of coastal deposits in the upper Bingmagou Formation in Zhulan village.

## Conclusions

The nature of the Precambrian sedimentary environment is largely determined based on changes in petrologic characteristics and sedimentary structures, namely on the theoretical inference and verification provided by sedimentology and stratigraphy. Obviously, experiments and tests of characteristics such as grain size, typical elemental content and mineral characteristics can also be useful for identifying the palaeoenvironment.

The Bingmagou Formation, which overlies NCC crystalline basement or the Palaeoproterozoic Xionger Group, is not older than 1.75 Ga and represents the beginning of stable deposition after the extension of the southern margin of the NCC and perhaps even the sedimentary response to the beginning of the Columbia Supercontinent breakup. With the formation and development of the Xionger rift trough on the southern margin of the NCC and subsequent continuous marine transgression, the depositional environment of the Bingmagou Formation changed from terrestrial alluvial fan to a marine gravelly and sandy coast. However, under the influence of local uplifts east of the Xionger rift trough and the terrain of the southern margin of the NCC being lower in the south and higher in the north, the alluvial fan deposits of the Bingmagou Formation only occur locally in the Shaoyuan, Mianchi, Lushan and Songshan regions. The gravelly and sandy coastal deposits that formed during the later transgression cover the entire area but developed first in the Luanchuan region at the very southern margin of the NCC, where the sedimentary environment was already marine when the Bingmagou Formation alluvial fans were still developing elsewhere. This region transitioned into a deeper-water environment in the later Yunmengshan period, when a carbonate ramp slope formed. The Bingmagou Formation did not develop in the Yangcheng region, where the Yunmengshan Formation directly overlies the NCC metamorphic basement or the Xionger Group volcanic rock. In conclusion, a highly distinctive type of environmental change gave rise to the unique depositional features of the Bingmagou Formation.
